# Assembly: A Key Enabler for the Construction of Superior Silicon‐Based Anodes

**DOI:** 10.1002/advs.202203162

**Published:** 2022-08-31

**Authors:** Miaomiao Jiang, Junliang Chen, Yingbing Zhang, Nan Song, Wan Jiang, Jianping Yang

**Affiliations:** ^1^ State Key Laboratory for Modification of Chemical Fibers and Polymer Materials College of Materials Science and Engineering Donghua University Shanghai 201620 China; ^2^ State Key Laboratory of Chemical Engineering East China University of Science and Technology Shanghai 200237 China; ^3^ Institute of Functional Materials Donghua University Shanghai 201620 China

**Keywords:** assembly mechanisms, assembly methodologies, lithium‐ion batteries, nanocrystallization and compound strategies, silicon‐based anodes

## Abstract

Silicon (Si) is regarded as the most promising anode material for high‐energy lithium‐ion batteries (LIBs) due to its high theoretical capacity, and low working potential. However, the large volume variation during the continuous lithiation/delithiation processes easily leads to structural damage and serious side reactions. To overcome the resultant rapid specific capacity decay, the nanocrystallization and compound strategies are proposed to construct hierarchically assembled structures with different morphologies and functions, which develop novel energy storage devices at nano/micro scale. The introduction of assembly strategies in the preparation process of silicon‐based materials can integrate the advantages of both nanoscale and microstructures, which significantly enhance the comprehensive performance of the prepared silicon‐based assemblies. Unfortunately, the summary and understanding of assembly are still lacking. In this review, the understanding of assembly is deepened in terms of driving forces, methods, influencing factors and advantages. The recent research progress of silicon‐based assembled anodes and the mechanism of the functional advantages for assembled structures are reviewed from the aspects of spatial confinement, layered construction, fasciculate structure assembly, superparticles, and interconnected assembly strategies. Various feasible strategies for structural assembly and performance improvement are pointed out. Finally, the challenges and integrated improvement strategies for assembled silicon‐based anodes are summarized.

## Graphical abstract

This review outlines the latest progress of silicon‐based assembled anodes from the aspects of assembly strategies, emphasizing the mechanism of the functional advantages for assembled structures. These summaries further deepen the understanding of the assembly mechanism and provide the systematic theoretical basis for the construction of superior silicon‐based anodes.

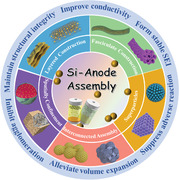

## 1 Introduction

**Figure 1:**
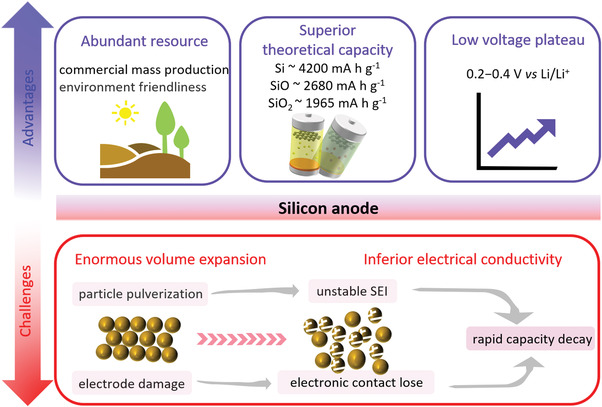
Summary of the main advantages and challenges of the silicon anode.

Substantial efforts have been committed to developing rechargeable lithium‐ion batteries with high energy density and long cycle life, to meet the explosive market demand from new portable electronic devices to large energy vehicles.^[^ ^1^, ^2^ ^]^ Lithium‐ion batteries occupy an important position in the energy storage market and have won the Nobel Prize in Chemistry in 2019.^[^ ^3^, ^4^, ^5^ ^]^ Graphite is the most common commercial anode material for lithium‐ion batteries, while the limited theoretical capacity (372 mA h g^–1^) cannot meet the mileage required for energy devices. Silicon, attributed to the low working potential (0.5 V vs Li/Li^+^), high theoretical capacity (4200 mA h g^–1^) and the natural abundance, has become one of the most promising anode materials to replace graphite for high energy lithium‐ion batteries.^[^ ^6^, ^7^, ^8^, ^9^ ^]^ However, the born defects of silicon anodes, including inferior electrical conductivity, sluggish lithium‐ion diffusion coefficient and serious volume expansion (>300%) during lithium insertion and extraction, easily increase electrode internal stress which will result in pulverization of electrode and the formation of unstable solid electrolyte interfaces (SEI) film, leading to rapid capacity decay (**Figure** **1**). To overcome these obstacles, a series of effective measures have been taken. First of all, the nanocrystallization and compound strategies of silicon‐based anode materials are developing vigorously, exploring various anode composites with different morphology and structure, including porous silicon, hollow nanospheres, silicon nanowires and so on, which further improve the utilization rate of active substance silicon during cycling.^[^ ^10^, ^11^, ^12^, ^13^ ^]^ In addition, the construction of silicon‐based anodes with the multistage complex structures is also popular, such as core‐shell structure, yolk‐shell, cage structure, layered structure, etc., which can further alleviate the volume expansion and other defects of silicon‐based anodes through the synergistic effect of hierarchical structures.^[^ ^14^, ^15^, ^16^ ^]^ However, it is still impractical to completely replace the graphite owing to the bottleneck of limited energy density and insufficient electrode loading. United States Department of Energy has proposed to increase the energy density up to 500 Wh kg^–1^ in commercial applications in the future. The improvement of the energy density mainly depends on the increase in the content of active material and the specific capacity of electrode material. Hence, novel silicon‐based electrode materials with high energy density have become a major focus of research and innovation, except for the optimization of the cell structure.^[^ ^17^, ^18^, ^19^, ^20^ ^]^

To effectively achieve the goal of high energy density for composite electrodes, a strategy has been proposed for assembling silicon‐based nanomaterials into micron‐sized hierarchical structures.^[^ ^21^, ^22^, ^23^, ^24^ ^]^ Assembly is a behavior in which basic structural units (molecules, nano materials, micron materials, etc) form a stable hierarchical architecture with regular geometric appearance based on the interaction of non‐covalent bonds.^[^ ^25^, ^26^, ^27^ ^]^ The assembly can significantly enhance the comprehensive properties of the material compared with the primary structural units, thus the assembly strategy is widely used in the fields of optoelectronics, biological medicine, catalysis, energy storage and so on.^[^ ^28^, ^29^, ^30^, ^31^, ^32^, ^33^, ^34^, ^35^, ^36^, ^37^ ^]^ As an anode material for lithium‐ion batteries, the assembled micron‐scale material has a lower interfacial area compared with the nanoparticles, which can effectively reduce the interparticle resistance at the nano‐size and mitigate the risk of side effects, thereby improving the energy density of the lithium‐ion battery. Moreover, under the same mass loading, the micron‐scale assembly can obtain a higher tap density, which makes the electrode thickness thinner and the electron transfer pathway shorter and increases the volumetric specific capacity of the whole cell.^[^ ^38^, ^39^, ^40^, ^41^, ^42^, ^43^, ^44^, ^45^ ^]^ Therefore, the assembly of silicon‐based nanomaterials can integrate the advantages of both nanomaterials and microstructures for the development of novel energy storage devices, which is an important strategy for the construction of silicon anode materials for lithium‐ion batteries with high energy density and excellent electrochemical properties.

The research focus of high‐performance silicon‐based anodes mainly tends to be the construction of hierarchical complex structures, and the introduction of assembly strategies is crucial for the fabrication of high‐performance silicon‐based structures with different morphologies and functions. In recent years, there has been more and more research on silicon‐based assembled anodes for improving the performance of batteries.^[^ ^46^, ^47^, ^48^, ^49^ ^]^ However, there is still a lack of a summary of silicon‐based assembled anodes, which fails to provide a better understanding of the assembly process. This review systematically summarizes the recent research advances in utilizing assembly strategies to address a series of challenges caused by the enormous volume variation and inferior electrical conductivity of silicon electrodes, emphasizing the advantages of silicon‐based assembled structures in the electrochemistry of lithium‐ion batteries. The driving forces, methods, influencing factors, and characteristics of the assembly process are also discussed to deepen the understanding of the assembled mechanism, and it is found that various assembly processes manipulated through interactions show surprising universality and great potential. Finally, the current challenges and development prospects of silicon‐based assembled anodes for lithium‐ion batteries are summarized.

## 2 Fundamentals of Assembly

The assembly behavior of basic structural units is mainly realized based on the interaction force of non‐covalent bonds. Various materials can be synthesized as assemblies with different forms and functions, depending on the characteristics of the basic structural unit, such as the morphology, shape, potential and functional groups on the surface, and the final structure with the lowest free energy after assembly. In this section, the driving force, methods and influencing factors for assembly, and the advantages of the assembled structure as the anode of lithium‐ion battery are discussed in detail.

### 2.1 Driving Force of Assembly

**Figure 2:**
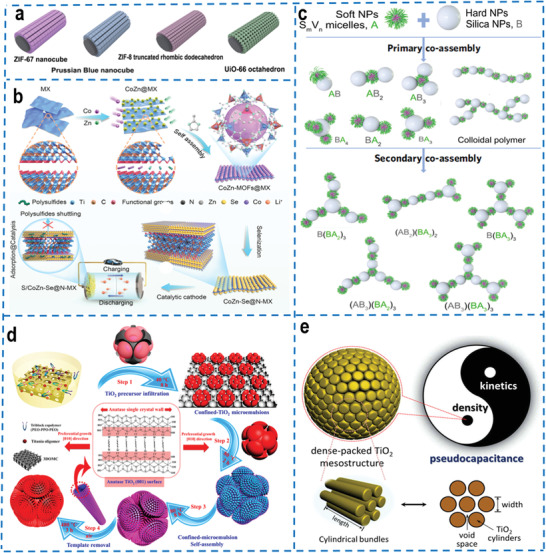
a) Self‐assembly of various types of colloidal MOFs on carbon cloth (Cobalt‐based ZIF‐67 nanocubes, Fe‐based Prussian Blue nanocubes, Zn‐based ZIF‐ 8 truncated rhombic dodecahedron and Zr‐based UiO‐66 octahedron). Reproduced with permission.^[^ ^58^ ^]^ Copyright 2021, Elsevier. b) Synthesis process illustration of CoZn‐Se@N‐MX. Reproduced with permission.^[^ ^59^ ^]^ Copyright 2021, Wiley‐VCH. c) Schematic illustration of primary co‐assembly and secondary co‐assembly of S_m_V_n_ micelles (S_m_V_n_ = PS_m_‐b‐ P2VP_n_, denoted as A) and silica NPs (denoted as B) in different feeding ratios to generate typical colloidal architectures. The valence of the co‐assembled structure can be precisely regulated by the relative size and feeding ratio of SV micelles and silica NPs. The PS core‐forming and the P2VP corona‐forming blocks are indicated by pink and green colors, respectively. Reproduced with permission.^[^ ^63^ ^]^ Copyright 2021, Springer Nature. d) Schematic representation of the formation process through confined‐micromulsion self‐assembly process (3D mesoporous bouquet‐posy‐like TiO_2_ Superstructures). Reproduced with permission.^[^ ^72^ ^]^ Copyright 2017, American Chemical Society. e) Schematic illustration of the 3D mesoscopic TiO_2_ design in pseudocapacitive charge storage. Reproduced with permission.^[^ ^73^ ^]^ Copyright 2021, American Chemical Society.

The transition of the materials from the dispersed state to the condensed state is the beginning of assembly, followed by the formation of the hierarchical assembly with a stable structure under the combined action of various forces. The internal driving forces determine the structure and performance of the assembled materials, including van der Waals force, hydrogen bond, electrostatic force and other non‐covalent bond forces, which are not a simple superposition of weak forces (such as atoms, ions, and molecules), but a kind of synergistic effect with holistic and complex.^[^ ^50^, ^51^, ^52^, ^53^, ^54^, ^55^, ^56^, ^57^ ^]^ Among them, electrostatic drive, a common non‐covalent interaction form, can be divided into attractive force and repulsive force according to the difference in the charges of particles. The electrostatic repulsion of components with the same charge can prevent agglomeration between components. On the contrary, electrostatic attraction can directly assemble components with opposite charges. For example, cationic‐modified colloidal metal‐organic frameworks (MOFs) were assembled onto negatively charged carbon‐based substrates driven by electrostatic attraction to form yolk‐shelled microcages.^[^ ^58^ ^]^ The proposed assembly strategy driven by electrostatic force has greatly expanded the application of MOFs, and the different types of MOFs (colloidal Co‐ZIF‐67, Fe‐Prussian blue (PB), Zn‐ZIF‐8, and Zr‐UiO‐66) can be successfully assembled on the surface of carbon cloth and carbon paper (**Figure** **2**a). Another research reported that bimetallic selenides on nitrogen‐doped MXene (CoZn‐Se@N‐MX) effectively immobilize and catalytically convert lithium polysulfide intermediates in lithium–sulfur batteries based on the assembly of MOFs and MXene via electrostatic interactions (Figure 2b).^[^ ^59^ ^]^

Van der Waals forces, another common non‐covalent interaction form, has also been widely used in the assembly strategy. For instance, nanowires and nanorods are uniformly and controllably assembled on the 2D MXene (Ti_3_C_2_) nanosheets through van der Waals forces, which favor the reduction of the free energy of the system and form a nano heterostructures.^[^ ^60^ ^]^ Van der Waals forces can also cooperate with other forces for the self‐assembly of nanorods through side‐by‐side, face‐to‐face, and end‐to‐end, respectively.^[^ ^61^ ^]^ Van der Waals forces, distinct from the electrostatic forces, bring nanocomponents to assemble together in an attractive force form, which requires the suitable solvents and ligands to achieve directional assembly with different dimensions. It also demonstrated the universality and large‐scale production of van der Waals force‐driven strategy.

Furthermore, the hydrogen bond is a driving force formed by the strong interaction of positively polarized hydrogen atoms with the negative charges of neighboring atoms.^[^ ^62^ ^]^ Cui et al. proposed a controlled co‐assembly strategy of soft (polystyrene‐b‐poly(2‐vinylpyridine) spherical micelles) (SV) and hard (silica nanoparticles) nanoparticles, which induced the preparation of colloidal assemblies with controlled valence characteristic via the hydrogen bonding between the pyridine groups in the poly(2‐vinylpyridine) (P2VP) micellar corona and the surface hydroxyl groups of silica nanoparticles.^[^ ^63^ ^]^ A series of colloidal assemblies with different saturated valence states and multidimensions (for instance, one‐dimensional (1D) chains, two‐dimensional (2D) networks, three‐dimensional (3D) blocks, etc.) were synthesized by adjusting the relative size and feeding ratio of the two particles, which could achieve the dynamic regulation for the valence state (Figure 2c). Moreover, the polymers and colloidal assemblies formed by the primary assembly can also generate secondary co‐assembly, which construct more complex hierarchical colloidal superstructures. The applicability of this assembly strategy for other functional particles is also investigated, and shows that gold (Au) nanoparticles, ZIF‐8 nanoparticles, polydopamine (PDA) nanoparticles, and core‐shell particles with silica shell could be controlled assembled with micelles to form numerous colloidal superstructures with controllable valence states. In addition, the authors successfully introduced the soft‐hard assembly strategy to the interface, realized the controllable assembly of colloidal molecules on the surface, and prepared colloidal brushes onto the micellar anchor via a “grafting‐to” method.

Magnetic force, a special driving force, can also synthesize the assembly with unique properties. Under the driving of a specific local magnetic field, the nanoparticles can be assembled into nanochains, nanorods and nanowires, mainly overcoming the thermal motion and electrostatic repulsion between particles. For example, 1D magnetic mesoporous silica nanochains with core‐shell structure is designed via a simple and reliable magnetic field guided interface co‐assembly method. Magnetic nanomaterials have high magnetic susceptibility to external magnetic fields, which are easy to align and rotate.^[^ ^64^ ^]^ The magnetic field‐assisted synthesis method can be extended to synthesize various nanochains with different compositions, sizes, shapes, or functions. In addition, there are some drives that can be applied to a larger scale, such as hydrophobic force, surface tension and capillary force drives.

### 2.2 Methods and Influencing Factors for Assembly

The assembly technology usually takes advantage of the characteristics of the initial structural unit, or modified functional groups on the surface of the initial structural unit, forming the hierarchical structure with a certain order based on certain rules. A series of mature preparation methods for assembled structure have been developed, including solvent evaporation induced assembly, microemulsion method, template‐assisted assembly method, chemical vapor deposition, electrospinning technique, etc.^[^ ^65^, ^66^, ^67^, ^68^ ^]^ Among them, the solvent evaporation induced assembly method mainly combines surfactant and sol‐gel method for fabricating nanostructures. The solvent volatilizes on the non‐volatile solid or liquid surface, which induces the assembly of molecules and nanoparticles into various arrays with complex and regular.^[^ ^69^ ^]^ Solvent evaporation induced nanoparticle assembly not only enables simple and efficient rapid synthesis, but also creates superstructures with different functions and morphologies, which involves the synergistic interaction of multiple driving forces. Long‐range ordered 3D Fe_3_O_4_ superparticle microspheres were obtained by solvent evaporation assembly strategy, based on a stable oil/water emulsion system.^[^ ^70^ ^]^ Ultra‐small few‐layer molybdenum disulfide (MoS_2_) were embedded into 3D honeycomb macro‐micro‐mesoporous carbon via a simple and cost‐effective aqueous evaporation‐induced assembly process.^[^ ^71^ ^]^ Of course, there are many influencing factors in the synthesis process of this method that need to be finely controlled, including surfactants, volatile components, precursor types, relative humidity, etc.

The microemulsion assembly method can limit the nucleation, growth, coalescence and agglomeration of nanoparticles, and precisely control the particle size and stability of nanomaterials, which has been widely adopted to construct secondary microspheres. Zhao and co‐workers proposed a confined microemulsion self‐assembly approach and demonstrated a 3D highly ordered mesoporous TiO_2_ superstructure with single‐crystal walls, bouquet‐posy‐like topologies, and radially oriented mesochannels, which consists of 1 spherical core and 12 symmetric satellite hemispheres (Figure 2d).^[^ ^72^ ^]^ It is found that the symmetry and complexity of the superstructure can be accurately manipulated by controlling the content and size of TiO_2_ precursor emulsion droplets. Recently, this research group has also designed a dense‐packed mesoscopic TiO_2_ mesoporous anode with accurately controlled mesoporous frameworks, in which the cylindroid TiO_2_ nano‐crystals are radially arranged from the sphere center, which is a promising anode material (Figure 2e).^[^ ^73^ ^]^ Similarly, mesoporous carbon spheres with dendritic‐like 3D radially aligned open mesochannels have been successfully synthesized through an emulsion‐induced interface assembly strategy.^[^ ^74^ ^]^ During the synthesis process, the interface assembly process of emulsions can be precisely controlled by simply adjusting the amount of dopamine and ammonia water, which constructs various mesoporous carbon spheres, including walnut‐like spheres, dendritic 3D radially oriented mesoporous spheres, vesicles, and so on.

**Figure 3:**
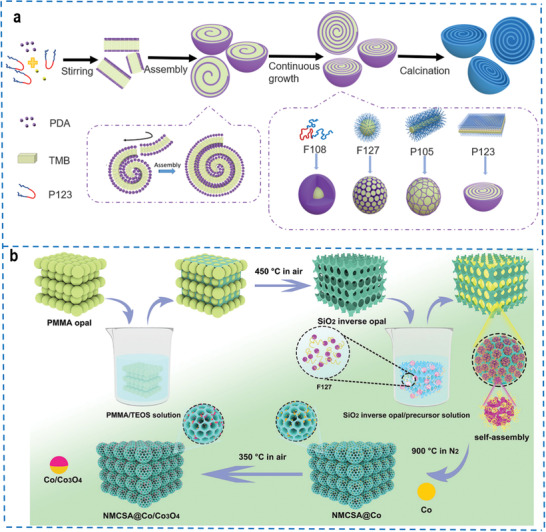
a) Schematic representation of the formation process of the spiral MCNs. A series of mesoporous carbon nanospheres were prepared using various triblock copolymers with different hydrophobic/hydrophilic ratios as templates: F108, F127, P105, and P123. Adapted with permission.^[^ ^79^ ^]^ Copyright 2021, American Association for the Advancement of Science. b) Schematic illustration of the preparation process for NMCSA@Co/Co_3_O_4_. Reproduced with permission.^[^ ^80^ ^]^ Copyright 2021, Wiley‐VCH.

Template‐assisted assembly method is a very efficient way to construct ideal hierarchical architectures that can utilize any surface modulated substrate as a scaffold (silica particles, metal‐organic frameworks, carbon spheres, metal particles, block copolymers, etc.), assisting the assembly of various particles in the appropriate active site arrangement.^[^ ^75^, ^76^, ^77^, ^78^ ^]^ It can be divided into the soft template method and hard template method according to the characteristics of the template itself and the different limiting abilities. Peng et al. successfully constructed multi‐shelled mesoporous carbon nanospheres (MCNs) with unique chirality through a lamellar micelle spiral self‐assembly method using block copolymer as the soft template (**Figure** **3**a). The mechanical interaction between the bending energy within the layers and the layer‐to‐layer interactions is the basis for the construction of helical structures with amphiphilic surfactants, which can balance the interaction for spiral self‐assembly.^[^ ^79^ ^]^ Taking the adjustment of the hydrophilic/hydrophobic ratio of the triblock copolymers as the clue, and the packing parameter of surfactants as the theoretical basis, various mesoscopic structures were constructed by using different templating agents. Among them, single cavity smooth nanospheres are obtained using Pluronic F108 as a template, radially oriented mesoporous nanoparticles are obtained using Pluronic F127 as a template, flower‐like mesoporous nanospheres are obtained using Pluronic P105 as a template, and spiral multi‐shelled mesoporous nanospheres are obtained using Pluronic P123 as a template. Besides the independent soft template assembly method, N‐doped mesoporous carbon sphere array composite (NMCSA@Co/Co_3_O_4_) can also be synthesized by a dual‐templates assembly method, in which the macroporous silica inverse opal is employed as the hard template and amphiphilic triblock copolymer Pluronic F127 is employed as the soft template (Figure 3b).^[^ ^80^ ^]^

Chemical vapor deposition (CVD) is the process of depositing vaporous substances on the gas phase or gas‐solid interface. The CVD strategy is widely used in various fields, due to its ability that effectively and finely control the structure of the final product by adjusting the precursor source, deposition temperature, and reaction rate. A nitrogen‐doped carbon layer was coated on the silicon nanoparticles by the CVD method, which maximized the stress release in the lithiated silicon and effectively relieved the volume expansion of the silicon nanoparticles during the (de)lithiation process.^[^ ^14^ ^]^ Cho et al. proposed a strategy that the silicon nanolayer and pyrolytic carbon were uniformly wrapped on the graphite substrate via the two‐step CVD process, which solved the problem of side reactions caused by structural failure.^[^ ^81^ ^]^ Moreover, this strategy resulted in uniform and dense coatings that could be precisely controlled for efficient assembly of building blocks. Compared with the CVD strategy, the assembly via the electrospinning strategy was much cheaper and more controllable.^[^ ^82^ ^]^

In addition to assembly method mentioned above, guided by a site‐selective liquid bridging interaction, the unique patch‐like particles assemble by heteroepitaxial growth into MOF‐based supra‐framework or supra‐colloidal with unprecedented precision.^[^ ^83^ ^]^ A high‐density corn‐like Co_3_O_4_/graphene assembly material was fabricated by in situ thermal treatment self‐assembly method.^[^ ^84^ ^]^ A polymer composite with fully exfoliated and highly aligned MOF nanosheets was prepared via layer‐by‐layer assembly.^[^ ^85^ ^]^ All the self‐assembly methods mentioned above can effectively realize the controllable self‐assembly of materials.

The control of the assembly process relates to various influencing factors, mainly including the concentration, size, physicochemical properties of self‐assembled units, types of solvents and surface additives, evaporation rate, temperature, and the surface physical and chemical properties of the substrate.^[^ ^86^, ^87^, ^88^, ^89^ ^]^ Chemical modification with organic molecules is considered to be a very important prerequisite for the realization of particle assembly. The organic molecules wrapped in the outer layer of the substrate play the dual roles of stabilizing particles and providing inter‐particle interactions. Through the interaction between these organic molecules, nanoparticles can be easily chemically assembly into aggregates with new structures.

### 2.3 Advantages of Assembly as Anode of Lithium‐Ion Battery

The excellent properties of nanoscale assembly can be regulated by simple manipulation or adjusting scale and geometric appearance. Therefore, the research of controllable functional nano‐assembly is one of the focuses of nanotechnology development at present and even for a long time in the future. For instance, uniformly sized hedgehog particles assembled from polydisperse nanoparticles have been successfully synthesized, thereby obtaining a series of assemblies such as nanorods, nanorods aggregates, hedgehog particles and low‐corrugation particles by controllably adjusting the reaction temperature, times, solvent, etc.^[^ ^90^ ^]^ After assembling molecules or nanoparticles into 1D, 2D, or 3D structures, the novel overall synergistic characteristics can be obtained. The structure of assembled molecules is highly versatile and can be designed by controlling the interactions between the original particles according to the required properties. The introduction of assembly technology into lithium‐ion batteries will greatly improve and expand various electrochemical properties.^[^ ^91^, ^92^, ^93^, ^94^ ^]^ On the one hand, after the assembly of nano‐materials, the composite of various conductive substances creates abundant active sites, and improves the electrical contact between the loose particles, further improving the conductivity of the material and promoting the rapid transmission of electrons.^[^ ^95^, ^96^ ^]^ Gao et al. have optimized the self‐assembly of SnO_2_ with MOF and graphene and prepared a unique structural configuration with abundant accessible electroactive sites for lithium ions storage, which shortened the ion transport pathways and exhibited superior electronic conductivity.^[^ ^97^ ^]^ Compared with monodisperse materials, the assembly structures exhibit higher electrochemical activity, faster charge transfer kinetics, and lithium ions diffusion.

On the other hand, the assembled nanomaterials have higher tap density, thinner electrode and shorter electron transport pathway under the same mass loading, resulting in a higher volumetric specific capacity.^[^ ^98^ ^]^ At the same time, the assembly can also reduce the resistance caused by the nano‐scale loose particles, and its porous structure can effectively alleviate the volume expansion during the charging and discharging process. Compared with loose nanoparticles, the assembly can achieve a lower interfacial area, effectively inhibit the excessive growth of SEI, reduce the risk of side reactions, and further improve the energy density of the lithium‐ion battery. Here, the assembly (Mn_3_O_4_@CNT/TiO_2_) of Mn_3_O_4_ nanoparticles with a stretchable 3D Hoberman spherical carbon nanotube (CNT) network not only takes advantage of the carbon nanotube materials to achieve highly conductive and enhances the reaction kinetics of Mn_3_O_4_ through electron density enhancement effect, but also introduces internal void space to adapt to the volume expansion of Mn_3_O_4_ during lithium insertion and extraction and maintains a stable outer surface of the material.^[^ ^99^ ^]^ The extra coating of the ultra‐thin and uniform TiO_2_ shell on Mn_3_O_4_@CNT/TiO_2_ can effectively limit the generation of SEI and improve stability. As an anode material for lithium‐ion batteries, the Mn_3_O_4_@CNT/TiO_2_ exhibits excellent rate capability, better cycling stability and a commercial‐level areal capacity. Other than that, the graphene‐wrapped porous Fe_3_O_4_/N‐doped C frameworks were successfully synthesized to keep structural integrity while preventing the direct contact between the active material and the electrolyte and reducing the formation of unstable SEI layer.^[^ ^100^ ^]^ The hierarchical porous structure can not only adapt to the volume change during cycling but also promote the penetration of the electrolyte.

## 3 Structural Design of Silicon‐Based Assembly

The combination of the nanoscale of silicon with features such as hollow porosity can be an effective way to solve the volume expansion of silicon anodes. However, the large specific surface area and high porosity caused by the porous structures lead to the low tap density and volumetric energy density of a single nano‐silicon structure. There are many research show that the silicon‐based assembly and even super‐structures can effectively solve the challenges of tap density and volumetric energy density. Whereas, the long‐term cycling stability of electrodes is still restricted by many factors, such as the unavoidable side reactions of the exposed silicon assembly in the electrolyte leading to the uncontrollable growth of the SEI film on material surface, and the low intrinsic electron and ionic conductivity of single silicon material, which seriously hinder its practical application. In recent years, these limitations have been effectively overcome by designing silicon‐based assembly with different hierarchical structures, such as the adoption of the surface and interface engineering, composite of single silicon with other materials, construction of different structures, doping of elements and other strategies, to further obtain silicon‐based composite assemblies with high energy density and long cycle life.^[^ ^101^, ^102^, ^103^, ^104^ ^]^ In this section, the silicon‐based assemblies will be briefly summarized and discussed, from the perspective of the methodologies and functions.

### 3.1 Spatially Nanoconfined Assembly

The large volume variation of silicon is inevitable during the repeated lithiation and delithiation process. To achieve the required high energy density, the volume change of silicon would become more serious when the electrodes with high mass loading. The nanocrystallization strategy of silicon units can effectively alleviate the volume expansion and pulverization of particles, but consequent instability and agglomeration have become a new problem. Furthermore, the large exposure of nano‐silicon particles would exacerbate the excessive reaction of the silicon particles with the electrolyte to form an unstable SEI film, resulting in lower Coulombic efficiency and rapid capacity decaying.^[^ ^105^, ^106^, ^107^ ^]^ The spatially nanoconfined assembly strategy can effectively resolve the agglomeration and instability of silicon‐based units by encapsulating silicon nanoparticles into fixed confined spaces to form independent structures.^[^ ^108^, ^109^ ^]^ The silicon nanoparticles were isolated through the external nanoconfined framework, which suppresses the excessive reaction between silicon and the electrolyte, and facilitates the formation of a stable and uniform SEI film, improving the performance of the battery. At the same time, the external nanoconfined framework can relieve the mechanical stress caused by the repeated lithium insertion and extraction of the silicon‐assembled electrode to a certain extent, and minimize the volume expansion.^[^ ^110^, ^111^, ^112^, ^113^ ^]^

**Figure 4:**
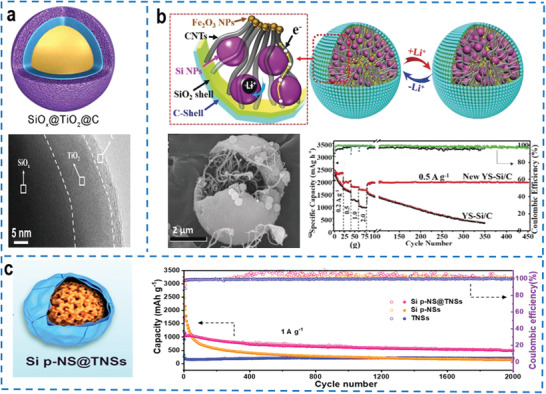
a) Schematic illustration and corresponding scanning electron microscopy (SEM) image of SiOx@TiO_2_@C. Reproduced with permission.^[^ ^116^ ^]^ Copyright 2020, Elsevier. b) Schematic illustration, SEM image and cycling performance of the YS‐Si/C electrode. Reproduced with permission.^[^ ^117^ ^]^ Copyright 2019, Wiley‐VCH. c) Schematic illustration and long cycle performance of the Si p‐NS@TNSs composite at 1 A g^–1^ for 2000 cycles. Reproduced with permission.^[^ ^118^ ^]^ Copyright 2020, American Chemical Society.

Liu et al. first proposed spherical carbon‐coated silicon nanocomposites by the spatially nanoconfined assembly strategy to effectively avoid the agglomeration of uniformly distributed silicon nanoparticles by the outer amorphous confined carbon coating layers, which buffered the large volume fluctuation of silicon nanoparticles during the cycling process.^[^ ^114^ ^]^ However, this disordered amorphous carbon coating could not accommodate the interfacial stress caused by the large volume fluctuation and finally breakages over subsequent cycling, thus failing to deliver an ideal cycling life for batteries. Therefore, a robust interfacial microporous confined carbon coating system was designed on the surface of silicon nanoparticles, which can accommodate the volume variation of silicon nanoparticles without providing void space.^[^ ^115^ ^]^ Alternatively, a multicomponent‐coating can be introduced to improve the stability of silica‐based materials. Based on the TiO_2_ with high mechanical stability, a dual‐shell coating structure was used to encapsulate the silicon‐based materials (SiOx@TiO_2_@C) to relieve the mechanical stress during the lithiation/delithiation process (**Figure** **4**a), further improving the interface strength of the silicon‐based particles, which endowed the electrode with excellent cycling stability.^[^ ^116^ ^]^ To better control the volume expansion of silicon electrodes while maintaining excellent properties of the core‐shell structure, a unique high‐density yolk‐shell structure with void spaces was designed, constructed by a carbon‐coated rigid SiO_2_ shell to encapsulate multiple silicon nanoparticles, CNTs and embedded Fe_2_O_3_ nanoparticles (Figure 4b).^[^ ^117^ ^]^ In this construction, the void space between the silicon yolks and confined carbon‐coated rigid SiO_2_ shell provided sufficient space for the volume expansion of silicon, which was conducive to the formation of a homogeneous and compact SEI layer, endowing the electrode with long cycling stability (reversible capacity retention of 95% after 450 cycles). Moreover, CNTs acted as conductive “highway” to provide fast lithium‐ion and electron transport for the outer double shell and the inner silicon yolks, which improved the overall tap density and conductivity. Besides the yolk‐shell structure, a porous‐core framework could also be introduced to provide sufficient space for the volume expansion of silicon and avoid pulverization of particles (Si p‐NS@TNSs) (Figure 4c).^[^ ^118^ ^]^ The tightly confined wrapped form of the nanosheets not only inhibited the direct contact between the porous silicon nanospheres and the electrolyte, but more importantly, provided a larger contact interface between the two components for efficient interfacial electron transport.

**Figure 5:**
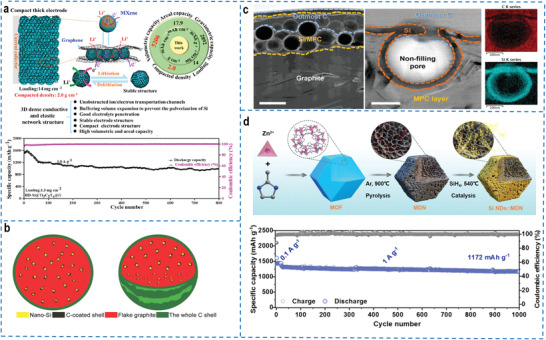
a) The microstructure, superior lithium storage mechanism and superior electrochemical performance of the compact thick HD‐Si@Ti_3_C_2_T_x_@G electrode. Reproduced with permission.^[^ ^120^ ^]^ Copyright 2022, American Chemical Society. b) Schematic illustration of the watermelon‐inspired Si/C microspheres. Reproduced with permission.^[^ ^122^ ^]^ Copyright 2017, Wiley‐VCH. c) The high‐resolution transmission electron microscopy (HRTEM) image of single C/Si@MPC‐G particle (left) and single pore in MPC layer (right). Scale bars: left ≈ 0.5 µm, right ≈ 100 nm. Reproduced with permission.^[^ ^123^ ^]^ Copyright 2020, Wiley‐VCH. d) Schematic illustration of the formation process and cycling performance (1 A g^–1^) of the zero‐strain high‐capacity silicon/carbon composite (Si NDs⊂MDN) enabled by a MOF‐derived space‐confined single‐atom catalytic strategy. Reproduced with permission.^[^ ^126^ ^]^ Copyright 2022, Wiley‐VCH.

Although the reserved hollow space design can accommodate the enormous volume change of the silicon anode during lithium insertion and extraction processes, the reserved space may exceed the demand for volume expansion, resulting in a series of problems with structural collapse and poor mechanical stability during the electrode calendaring process, which thus sacrifices the capacity and energy density of silicon‐based anodes. In response to this, wang et al. had fabricated a pressure‐resistant silicon structure by constructing a compact silicon shell coating on the porous secondary microparticles, which were microsilicon clusters composed of many silicon nanoparticles by a bottom‐up microemulsion method, followed by assembled to form a graphene‐encapsulated silicon‐shell‐protected silicon hollow structure.^[^ ^119^ ^]^ The dense and robust silicon coating could resist high pressures of over 100 MPa, which remained barely broken after the calendaring procedure and exhibited an impressive volume capacity of 2041 mA h cm^–3^. The graphene cage with excellent mechanical strength and flexibility combined with the internal void space of the micro‐silicon cluster, which promoted the silicon nanoparticles to expand within the graphene cage while maintaining interfacial stability and structural integrity during cycling. Another strategy, the integration of dense structural engineering and dually encapsulated silicon structure, was proposed and successfully synthesized a 3D high‐density Ti_3_C_2_T_x_ MXene and graphene double‐encapsulated silicon monolith structure (HD‐Si@Ti_3_C_2_T_x_@G) to ensure mechanical stability while achieving an ultrahigh volumetric capacity of 5206 mA h cm^−3^ and gravimetric capacity of 2892 mA h g^–1^ for lithium‐ion batteries (**Figure** **5**a).^[^ ^120^ ^]^ The 3D conductive and flexible network structure provided electron and ion transportation channels, effectively alleviating the volume change of silicon. The graphene with low content endowed the HD‐Si@Ti_3_C_2_T_x_@G with high electrical conductivity (151 S cm^–1^) and high density (1.6 g cm^–3^). The dual‐encapsulated confined strategy greatly improved the utilization rate of the silicon anode and ensured the stability of the electrode structure and propitious electrolyte penetration, exhibiting superior long‐term cycling stability.

Based on the spatially nanoconfined assembly of nano‐engineered silicon, the direct embedding of silicon into the confinement matrix has been also considered a reasonable strategy to improve the mechanical properties and achieve high energy density of silicon electrodes. Via a scalable microemulsion method, a unique nano/microstructured silicon‐carbon hybrid composite assembly was successfully synthesized by directly embedding nano‐scale silicon particles into micron‐sized amorphous carbon spheres coated with an external graphitic carbon coating layer.^[^ ^121^ ^]^ The confined double carbon structure tightly encapsulated silicon particles significantly reduced the absolute stress and strain during repeated cycling, and demonstrated high‐capacity retention of 80% and a fast charge‐discharge capability of 12 min. In addition to being able to conduct electricity quickly, the carbon skeleton assembly also maintained structural integrity after extremely long‐term cycling. Herein, a Si/C microsphere inspired by the structure of watermelon fruit was reported (Figure 5b) to alleviate the volume expansion and particle fracture under high mass loading.^[^ ^122^ ^]^ The carbon‐coated Si nanoparticles were uniformly dispersed in the flake graphite matrix by the spray drying assembly method, followed by the deposition of the whole carbon shell. Based on the dual protection strategy including optimized size distribution and hierarchical buffer confined structure, the Si/C anodes exhibited multiple advantages: i) the carbon‐coated nano‐silicon particles alleviated the tremendous volume variation, ii) the internal void space accommodated the volume expansion of nano‐silicon, iii) the confined graphite framework inhibited the aggregation of the nano‐silicon particles, iv) the whole confined carbon shell effectively prevented the exposure of nano‐silicon under high‐pressure density. The secondary assembly of nano‐silicon particles effectively increased the tap density by 220% relative to loose silicon nanoparticles, which was of great significance for the commercial application of high‐energy‐density battery electrode materials. In addition, graphite as a lubricant allowed the composite electrodes to endure high mechanical pressure during calendaring without rupture during electrode calendaring (Figure 5c), which was beneficial to obtaining high energy density at the battery pack scale.^[^ ^123^ ^]^

The nanocrystallization strategy of silicon units can effectively alleviate the volume expansion and pulverization of silicon particles during the repeated lithiation and delithiation process. The critical particle size (150 nm) of silicon units plays an important role in maintaining structural stability, according to the previous literature. The cracking and fracture behavior of silicon nanoparticles exhibits a strong size dependence upon the first lithiation: particle size below 150 nm, the particles neither cracked nor fractured during the first lithiation; above 150 nm, the particles initially formed cracks and then fractured due to lithiation expansion.^[^ ^124^, ^125^ ^]^ Therefore, silicon with feature sizes reduced to the sub‐nanometer scale can maximally tolerate the huge dimensional strains without fragmentation. Small‐scale silicon introduced into a limited space by a spatially nanoconfined assembly strategy will lead to more enhanced properties, showing good comprehensive performance and commercial viability. MOF is a well‐ordered porous material with adjustable porous channels structure. When assembled with small‐sized silicon, the nanopores channels or molecular cages of MOF can achieve uniform confinement distribution of small‐sized silicon and effectively prevent the agglomeration of silicon particles, giving full play to the advantages of both. For example, ultra‐small silicon nanodots can be embedded in MOF‐derived nanoreactors of porous carbon matrix by a novel MOF‐derived single‐atom catalyzing strategy, which synthesized a Si/C composite assembly material (Si NDs⊂MDN) with unique zero‐strain property and high lithium storage capacity (Figure 5d).^[^ ^126^ ^]^ The MOF‐derived porous carbon framework provides zero‐strain property and high structural stability for the assembly, and the ordered nanopores channel of MOF can better disperse ultra‐small silicon nanodots while providing a fast diffusion channel for lithium ions transport. The ultra‐small silicon nanodots within the nanopores provide more active sites, facilitating high lithium storage capacity and providing a novel silicon‐based anode material with long cycling life. Moreover, the subnano‐sized (<1 nm) silicon was embedded into a robust matrix to also effectively address the structural stability issues of porous frameworks, thereby exhibiting good cycling performance (capacity retention of ≈91% after 2875 cycles) and calendar life (97.6% for 365 days).^[^ ^127^ ^]^

### 3.2 Layered Structure Assembly

To overcome the low electrical conductivity and large volume variation of silicon electrodes, layered silicon‐based assemblies are designed by introducing highly conductive 2D structural materials. 2D structural materials with the high specific surface area can greatly shorten the pathways of electron transport and lithium‐ion diffusion, which achieve rapid insertion and extraction of lithium ions, reducing polarization and improving rate performance.^[^ ^128^, ^129^, ^130^, ^131^, ^132^ ^]^ Moreover, the gap of the layered structures can not only adapt to the mechanical stress accompanying the insertion and extraction of lithium ions, effectively relieve the volume expansion of the silicon‐based electrodes, but also significantly hinder the agglomeration of silicon particles and promote the stable and efficient cycling of lithium‐ion batteries.^[^ ^133^, ^134^ ^]^ Generally, there are two main strategies for layered silicon‐based assembly: silicon‐based particles or nanowires are directly assembled with 2D materials; silicon‐based thin films or nanosheets with 2D structures are directly assembled with other materials. For example, a nitrogen‐rich carbon/silicon composite with a graphene‐like structure was synthesized based on the electrostatic interaction between the carboxyl group and amino groups, which improved the electronic conductivity and effectively buffered the volume change of silicon nanoparticles during repeated cycling.^[^ ^135^ ^]^ Similarly, a sandwich‐like Si/Ti_3_C_2_T_x_ MXene composite was prepared by uniformly decorating silicon nanoparticles on loosely multilayered Ti_3_C_2_T_x_ with accordion‐like structure.^[^ ^136^ ^]^ The defects on the loosely multilayered Ti_3_C_2_T_x_ MXene flakes were conducive to the interfacial assembly of silicon nanoparticles with flakes, forming a 3D electronic transfer pathway, which promoted electron transport during cycling. In addition, combining with the inherent large surface area and good flexibility of MXene nanosheets, the larger interlayer distance of accordion‐like structured MXenes might be conducive to the flexible intercalation of Si‐based materials, providing an elastic buffer to accommodate the volume variation of silicon electrodes during lithiation.

The strategy that low‐dimensional silicon elements were embedded into 2D materials to construct a layered assembly structure can effectively alleviate the volume expansion of Si‐based electrodes while improving the conductivity.^[^ ^137^ ^]^ However, the exposed silicon elements between the layers may lead to the formation of unstable SEI films upon direct contact with the electrolyte, reducing the cycling efficiency and resulting in rapid capacity fading. To solve this problem, SiO_2_ nanoparticles could also be firmly anchored on the flexible MXene layers using the bonding effect and then synthesized SiO_2_/MXene microspheres by a spray drying method, which effectively reduced the specific surface area, relieved the side reactions and improved the Coulombic efficiency, thereby improving the structural stability during the long‐term cycling.^[^ ^138^ ^]^ However, this strategy cannot significantly address the defects of exposed silicon particles. Therefore, a new strategy has been proposed, which is to encapsulate the silicon particles with conductive carbon and metal to suppress the excessive contact between the particle silicon and the electrolyte, and then assemble the layered structure.^[^ ^139^, ^140^ ^]^ Conductive metallic shell and carbon shell could not only improve the conductivity and corresponding mechanical stress of the whole electrode, but also acted as a shelter to effectively prevent excessive contact between silicon and electrolyte, and alleviated the abnormal growth of SEI films. Additionally, the layered structure loading with evenly distributed silicon nanoparticles eliminated collisions between silicon nanoparticles during cycling, providing a buffer space for volume expansion. 2D layered materials can be used as a composite substrate or a 3D scaffold to be assembled with silicon to design and synthesize a series of 3D Si/C assembly materials.^[^ ^141^ ^]^ The constructed layered structure combines the structural features of both micro‐scale building blocks and nano‐scale assemblies, thus can significantly improve the charge transport‐ability, strain accommodation, and structural stability after assembly. In order to better alleviate the intrinsic defects of Si electrodes, a unique SiOC material with tunable chemical component, optimistic reversible capacity, multiple synthetic routes, and small volume expansion was selected to fabricate a 3D lamellar SiOC@C/graphene assembly with porous microstructure via electrostatic self‐assembly process and hydrothermal reaction.^[^ ^142^ ^]^ The SiOC was composed of amorphous SiO_x_C_4‐x_ tetrahedral units and disordered free carbon nanoclusters, which randomly distributed in the amorphous network and was difficult to form a consecutive conductive system, resulting in poor electrical conductivity. Thus, the graphene supporter, disordered free carbon nanoclusters in SiOC and the carbon layer on the surface of SiOC were synergistically assembled into a multi‐dimensional interconnected conductive system to avoid the aforementioned defects. The multidimensional conductive structure and the robust structure synergistically enhanced the electrical conductivity and promoted the rapid ion transport capability, exhibiting excellent rate capability as the anode electrode of lithium‐ion batteries, further enhancing the potential applications of SiOC. To overcome the drawbacks caused by this disordered distribution of carbon network distribution, Yang's group synthesized a new porous SiOC nanocomposite anode with a carbon matrix homogeneously distribution at the atomic scale to provide an excellent conductive network.^[^ ^143^ ^]^ And then, the porous SiOC nanospheres were encapsulated into a 2D conductive graphene film via boron doping induced self‐assembly, which reduced the interparticle resistance of nanospheres.^[^ ^144^ ^]^ The lamellate graphene skeleton structure released the volume change stress of SiOC, facilitated electrons and ions transfer pathways and improved the rate capability of the assembled anode, and also inhibited continuous growth of the SEI film to ensure interface stability during cycling.

**Figure 6:**
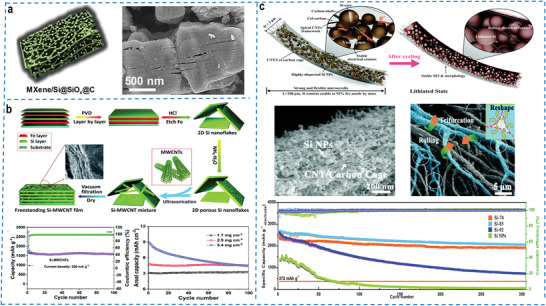
a) Schematic illustration and SEM image of MXene/Si@SiOx@C nanohybrids. Reproduced with permission.^[^ ^146^ ^]^ Copyright 2019, American Chemical Society. b) Schematic illustration of the fabrication process, cycling performance (based on an areal loading of 2.9 mg cm^–2^) and areal capacity (different areal loadings) of the freestanding Si‐MWCNT film. Reproduced with permission.^[^ ^149^ ^]^ Copyright 2020, Royal Society of Chemistry. c) Schematic illustration of one Si@CNT/C‐microscroll before and after electrochemical cycling, SEM images and cyclability (0.2 A g^–1^) of Si@CNT/C‐microscroll. Reproduced with permission.^[^ ^159^ ^]^ Copyright 2019, Royal Society of Chemistry.

Similarly, the introduction of an integral protective coating framework outside the silicon‐based lamellar structure can perfectly avoid the direct exposure of silicon‐based particles to the electrolyte.^[^ ^145^ ^]^ A class of MXene/Si@SiOx@C sandwich superstructure (**Figure** **6**a) with interfacial amorphous and auto‐adjustable layer space was reported for achieving high‐performance LIBs applications, by introducing Si and SiOx into MXene to construct a conductive network that was further covered with an N‐doped carbon coating layer.^[^ ^146^ ^]^ The N‐doped carbon coating layer perfectly limited the exposure of Si and formed a stable SEI film, which effectively alleviated the silicon‐based electrode volume expansion only to 12% combined with the layer‐stacked MXene framework, with no obvious fracture and pulverization after long cycling. More interestingly, under the synergistic effects of MXene, Si, SiOx, and N‐doped carbon coating layer, the rationally designed MXene/Si@SiOx@C sandwich superstructure with good electronic conductivity, rapid lithium ions mobility and flexible interlayer spacing was endowed with excellent structural stability and long‐term cycle stability.

There is no doubt that the introduction of 2D conductive materials can improve the electrical conductivity and the cycling stability of the electrode after careful structural design. Nevertheless, 2D nanosheets only can offer surface conductivity when composited with silicon, while the conductivity between silicon nanoparticles is still insufficient to achieve an effective transmission path, which is not conducive to the migration of lithium ions. Therefore, the MXene@Si/CNTs composites with a hierarchical interpenetrating conductive network were successfully synthesized by introducing 1D carbon materials, which could improve the electrical conductivity from point to point and on planes by shrunken MXene nanoflakes and CNTs that could connect silicon nanoparticles in series.^[^ ^147^ ^]^ The hierarchical interpenetrating conductive network realized the fast transport of lithium ions while maintaining the stability of the electrode structure, showing more excellent rate capability. Alternatively, 0D silicon nanoparticles, 1D CNTs and 2D Ti_3_C_2_T_x_ nanosheets were directly co‐assembled to construct flexible free‐standing and binder‐free composite films, which featured electrical conductivity, excellent mechanical flexibility, and unique hierarchical pore structure.^[^ ^148^ ^]^ In particular, both the restacking of Ti_3_C_2_T_x_ nanoflakes and the aggregation of silicon nanoparticles were efficiently inhibited. The reversible capacity of the composite film was 2.2 mA h cm^–2^ at an area mass loading of 2.26 mg cm^–2^, which had a promising application prospect for portable wearable devices.

In addition to the above mentioned that silicon‐based particles or nanowires are directly assembled with 2D materials, silicon‐based thin films or nanosheets with 2D structures can also be directly assembled with other materials to construct high‐energy‐density silicon‐based electrodes. In order to achieve the high areal capacity of freestanding silicon electrodes while maintaining a stable structure, a novel 2D porous amorphous silicon nanosheet was designed to form a freestanding Si‐MWCNT assembled films with a high silicon content via layered assembly with ultralong 1D multi‐walled carbon nanotubes (MWCNTs) with high mechanical strength (Figure 6b).^[^ ^149^ ^]^ The interconnected MWCNT network could not only endow layered assembled electrodes with fast and continuous electron transportation, but also prevent the restacking of adjacent silicon nanoflakes, providing the space to accommodate volume expansion. The abundant pore structure inside the layered assembled films effectively enhanced the diffusion of lithium ions and thus improved reaction kinetics. The compact and interconnected network structure formed by the entanglement of MWCNTs with silicon flakes exhibited an area mass loading of 5.4 mg cm^–2^, a high specific capacity of 1556 mA h g^–1^, and an areal capacity of 4.5 mA h cm^–2^. It was noteworthy that the full battery delivered a high gravimetric energy density of 484.7 Wh kg^–1^. Recently, the freestanding electrode has attracted tremendous attention due to its high energy density, good mechanical stability, safety, nonuse of conductive agent, binder, and current collectors.

### 3.3 Fasciculate Structure Assembly

Silicon‐based materials are introduced into the highly conductive 1D carbon materials to assemble fasciculate structures, which can provide a proposed radial short‐distance transport path for electrons and lithium ions, realizing rapid insertion and extraction of lithium ions and enhancing the rate capability of the assembled electrodes. Moreover, the abundant void space inside the fasciculate structure can accommodate the volume variation of silicon‐based particles.^[^ ^150^ ^]^ Silicon particles with good dispersibility are assembled with 1D carbon nanotubes under the electrostatic driving force to form fasciculate structures, or fasciculate fibers by electrospinning, both of which provide fast radial electron transfer paths and exhibit high‐rates capability and high electrical conductivity.^[^ ^151^, ^152^, ^153^ ^]^ However, the silicon particles assembled on carbon nanotubes are still unavoidably exposed in the electrolyte, likewise, there is not enough space within the fasciculate fibers to accommodate the volume expansion. Combined with the advantages of spatial confinement, carbon nanofibers are fabricated by encapsulating silicon nanoparticles with cavities, which allow the volume change of silicon nanoparticles, significantly improving the structural stability during cycling.^[^ ^154^ ^]^ Another strategy is to coat a silicon oxide/carbon hybrid layer on the surface of the silicon‐based building block, which is deposited onto a carbon nanotube to construct a three‐layer coaxial nanotube (SiO_2_/C@SiO_2_@CNT).^[^ ^155^ ^]^ Among them, the CNTs acted as a supporting framework and the outermost carbon layer served as double‐layer buffer media, which could buffer the mechanical stress caused by the volume variation and serve as a conductive matrix to provide excellent electronic conductivity for the material. The void spaces within the CNTs offered a swift and efficient route for the rapid transportation of electrolytes, and the synergistic effect and specific structure of the material modules endowed excellent cycling stability for the electrode.

To ensure the structural integrity of silicon electrodes in practical applications, a relatively low amount of silicon has to be used to alleviate the structural damage caused by volume expansion. Whereas, such a low silicon content seriously weakens the capacity advantage of the silicon‐based materials at the electrode level, which cannot reach the commercial level and seriously hinders the practical application of silicon‐based electrodes. On the premise of ensuring the structural stability, the electrode capacity was improved by increasing the silicon content in the material, and the coaxial fibers with a core‐shell structure (silicon content of ≈50 wt%) were synthesized by a dual nozzle electrospinning technique.^[^ ^156^ ^]^ The carbon fiber shell wrapped with silicon could effectively suppress the excessive contact between silicon particles and electrolyte, and the void space between the internal silicon particles can also buffer the volume expansion during cycling, endowing the electrode with an excellent initial capacity of 1384 mA h g^–1^ and outstanding cycle life (99% capacity retention after 300 cycles). However, the physical contact points among internal silicon particles or individual fibers are often insufficient, easily leading to high electrode contact resistance and prolonging charge transfer pathways through electrodes, which will impede its energy storage properties such as rate capability and power density. Therefore, the carbon conductive network can be introduced into the fasciculate fibers to improve the conductivity and stability of the electrode,^[^ ^157^ ^]^ Wu et al. encapsulated Si nanoparticles in a honeycomb‐like carbon framework and further wrapped by an interconnected cobweb‐like carbon‐shell network containing multi‐walled carbon nanotubes to prepare silicon/interconnected hollow carbon composite fibers with a hierarchical core‐shell structure.^[^ ^158^ ^]^ The interconnected carbon networks with high mechanical strength not only provided more access sites for electron transfer to improve the transport capacity of ions and electrons, but also effectively inhibited the penetration of electrolytes. Moreover, the honeycomb‐like pores inside the porous carbon framework effectively accommodated the volume expansion of silicon, resulting in the silicon‐based fiber electrode demonstrating excellent rate performance and cycling stability, and the original morphology and structure could still be maintained after 100 cycles.

Another interesting strategy was proposed by Wang et al. A novel Si@CNT/C ultralong topological microscroll (lengths > 100 µm) was synthesized by assembling Si‐anchored bundled CNTs onto cellulose nanosheets, followed by a freeze‐drying process to realize topological microscroll wrapping of cellulose nanosheets bunched Si@CNT (Figure 6c).^[^ ^159^ ^]^ The topological microscrolls formed by the rolling of cellulose nanosheets effectively confined the silicon nanoparticles in carbon nanotubes/carbon cages, which achieved an unprecedented silicon loading of 92%. The twining helical carbon nanotubes/carbon cage structure could stably support the uniformly dispersed silicon nanoparticles and form abundant voids to provide sufficient buffer space, which was beneficial to relieve the expansion pressure of the silicon particles. The binder‐free and free‐standing flexible electrode assembled from this microscroll exhibited an extremely high specific capacity of 2704 mA h g^–1^ and unparalleled cycling stability, maintaining a capacity of 2056 mA h g^–1^ after 300 cycles. This green and scalable synthesis method provided an efficient route for the commercial application of silicon anodes in lithium‐ion batteries.

### 3.4 Superparticles Assembly

**Figure 7:**
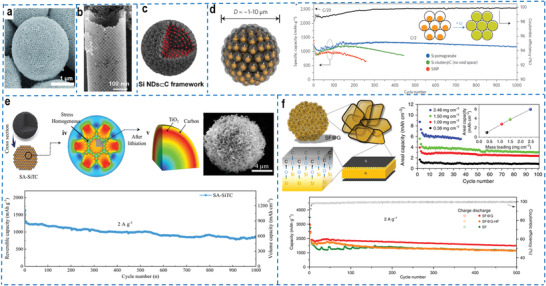
a) SEM image of the self‐assembled 3D plasmonic colloidosomes. Reproduced with permission.^[^ ^162^ ^]^ Copyright 2015, Wiley‐VCH. b) A high‐resolution SEM (HRSEM) image of a single Fe_3_O_4_ supertube. Reproduced with permission.^[^ ^163^ ^]^ Copyright 2019, Elsevier. c) Schematic illustration of the Si NDs⊂C framework. Reproduced with permission.^[^ ^164^ ^]^ Copyright 2019, Wiley‐VCH. d) Schematic illustration and reversible delithiation capacity (1000 galvanostatic cycles) of the silicon pomegranates. The rate is C/20 for the first cycle and C/2 for later cycles (1C = 4.2 A g^–1^ active material). Reproduced with permission.^[^ ^166^ ^]^ Copyright 2014, Springer Nature. e) Chemomechanical modeling of stress distribution during lithiation and reversible delithiation capacity of SA‐SiTC at 2 A g^–1^ (0.1 A g^–1^ for the first two cycles). Reproduced with permission.^[^ ^168^ ^]^ Copyright 2020, Wiley‐VCH. f) Schematic illustration, areal capacity at various active material mass loadings, and cycling performance over 500 cycles at 0.2 A g^–1^ (bottom) of SF@G. Reproduced with permission.^[^ ^170^ ^]^ Copyright 2020, Springer Nature.

It is well‐known that the realization of complex functions mostly needs to go through a multi‐scale hierarchical and orderly self‐organization/collaboration process from small to large. Generally, inorganic nanoparticles can spontaneously form various structures under certain conditions, whether ordered or disordered, which belong to the self‐assembly process.^[^ ^25^ ^]^ “Superparticle” is considered to be an ordered nano/microstructure formed from the self‐assembly of molecules and nanoscale building blocks under certain synergistic effects.^[^ ^160^ ^]^ For example, a carbon superstructure with rigid skeleton was synthesized by “rivet” links, reducing the adsorption energy, meliorating charge density distribution, enabling the high accessibility of interior protophilic sites, achieving efficient ion diffusion with a lower energy barrier, and providing the ultra‐stable charge storage and fast proton‐coupled dynamics.^[^ ^161^ ^]^ Based on a novel inverse microemulsion template strategy, a truly 3D ordered plasmonic colloidosomes (**Figure** **7**a) driven by the minimization of the total interface free energy was fabricated for the first time using monodispersed spherical nanoparticles as the basic building blocks.^[^ ^162^ ^]^ The controllable self‐assembly of particles into ordered superstructures from nano‐scale to macro dimensions had been achieved through precise positioning, which had high potential in both fundamental research and practical applications. The assembly of microsphere superstructure at all scales realized the efficient‐simple‐controllable construction of uniform microsphere superstructures, showing the highly tunable in morphology and size, as well as versatile for different types of functional building blocks, such as nanospheres, various macromolecules, and inorganic ionic compounds.^[^ ^160^ ^]^ The self‐assembly strategies, such as supramolecular interaction, controllable stacking, and induced assembly, can be used to achieve more precise control for the assembly at the nanoscale to obtain advanced functional assembly materials with more controllable structures, more diverse morphologies and better performance. When the self‐assembled superstructure is used as an electrochemically active material, its unique close‐packed structure can improve the tap density of the electrode and promote the transfer of ions and electrons, thereby improving the high energy density and long‐cycle stability of the electrode. A unique tubular superlattice was successfully synthesized and maintained a stable ordered structure over long cycles.^[^ ^163^ ^]^ The superlattice consisted of a single layer of close‐packed carbon‐coated Fe_3_O_4_ nanoparticles (Figure 7b), allowing the individual Fe_3_O_4_ nanoparticle to independently participate in electrochemical reactions. It can be seen that complex superstructure can actually exhibit more excellent electrochemical performance than simple nanostructures, which is of great significance for the development of high‐performance electrochemical devices. Therefore, the energy storage and conversion function of an electrode can be better improved by preparing the silicon‐based anodes with superstructure. Silicon‐based superstructure with a higher tap density can obtain thinner electrodes under the same mass loading, which effectively improves the volumetric specific capacity of the electrodes. Furthermore, the silicon‐based assembly with superstructure needs to be rationally designed to reduce the interfacial area of the electrode and avoid side reactions with the electrolyte, obtaining a higher Coulombic efficiency. It can also significantly reduce interparticle resistance and improve the overall energy density of the lithium‐ion battery. For example, silicon nanodots are uniformly super‐assembled into a carbon framework with high dispersity to synthesize spherical Si/C composites (Si NDs⊂C) with the hierarchical pore structure (Figure 7c).^[^ ^164^ ^]^ The silicon nanodots combined with the hierarchical pore structure are beneficial to high‐capacity, fast and stable lithium storage, and the Si NDs⊂C framework simultaneously exhibits low lithiation stress and excellent mechanical stability. Compared with loose silicon particles, the silicon‐based building blocks within the superstructure have better electrical contact and shorter electron transfer paths with each other, improving the rapid transport of charge during lithium insertion and extraction.

To alleviate the electrical contact loss and particle fracture that are considered to be the main factors for the Si capacity decay, a 3D binder‐free and free‐standing type anode electrode paper was well‐designed. A 3D interconnected nitrogen‐carbon network connected with hollow carbon nanospheres with uniformly distributed silicon nanodots (SHCM/NCF) to improve the structural stability and electrical conductivity of silicon‐based assembled anodes.^[^ ^165^ ^]^ The nitrogen/carbon network could maintain excellent electrical contact for rapid electron/ion transport, and the buffer layer inside the cross‐linked network could accommodate the volume expansion during cycling. The as‐fabricated SHCM/NCF paper anode exhibited a reversible capacity of 1442 mA h g^–1^ for 800 cycles. However, this intertwined structure still cannot avoid the contact between silicon and electrolyte. A novel pomegranate‐like silicon/carbon microsphere superstructure (Figure 7d) was designed and synthesized, introducing an internal void space and an electrolyte barrier layer, in which individual silicon nanoparticle was wrapped by a conductive carbon layer, and then encapsulated in a carbon bag.^[^ ^166^ ^]^ First, nano‐scale Si particles (80 nm) effectively prevented fracture, and the carbon framework served as both a mechanical backbone and an electron transport highway. The carbon encapsulation of entire secondary particles confined the SEI to the outer surface of microspheres instead of individual particles, which reduced the area that can be permeated by electrolyte and achieved a high Coulomb efficiency (99.87%), and reserved sufficient space for expansion/contraction following (de)lithiation. Benefiting from the space‐efficient packing inside the secondary silica particles, the tap density (0.53 g cm^–2^) was significantly higher than that of the randomly packed primary silica particles (0.15 g cm^–2^), exhibiting a higher volumetric capacity (1270 mA h cm^–3^). The unique structure endowed excellent cycling stability of batteries, maintaining 97% of the capacity after 1000 cycles.

Mechanical tensile stress with different degrees is generated during the lithiation process, which may destroy similar porous and hollow structures, resulting in poor mechanical properties and structural collapse of the electrodes. Therefore, mechanical tensile stress is required to be effectively controlled to maintain the electrode structure stability and excellent electrochemical performance. At present, the most common solution to control tensile stress is to coat the surface of the materials to maintain the mechanical stability of the structure.^[^ ^167^ ^]^ Shi and co‐workers established a finite element model for cross‐scale simulating the stress evolution of self‐assembled functional structures, and skillfully designed a scalable self‐assembled hierarchical Si@TiO_2_@C (SA‐SiTC) (Figure 7e) based on core‐shell Si@TiO_2_ nanoscale building blocks guided by an effective stress management model.^[^ ^168^ ^]^ The theoretical simulation combined with the experimental exploration showed that the TiO_2_ layer and carbon layer were regarded as buffers, and the interparticle interaction in the self‐assembly effectively reduced the tensile stress on the silicon surface and the risk of particle cracking, which comprehensively ensured the structural stability of the assembly. The surface‐coated carbon layer and TiO_2_ layer effectively limited the overgrowth of SEI while providing a fast electron diffusion path, and improving the Coulombic efficiency (initial Coulombic efficiency ≈ 80.9%), thus ensuring the long‐cycle stability of the electrode (842.6 mA h g^−1^ after 1000 cycles). More importantly, this self‐assembled structure with higher tap density exhibited a high volumetric capacity of 1174 mA h cm^–3^, and the outward tensile stress of internal Si and the specific capacity was 0.51× and 2.33× that of individual particles after 500 cycles, respectively, and remained integrity of the morphology during (de)lithiation. Therefore, the mechanical strength of the material is as essential as the electrochemical performance of the electrode, and both play an important role in the actual production process of the battery. For this purpose, a unique hierarchical carbon‐nanotube@silicon@carbon (CNT@Si@C) microsphere was designed and synthesized to fully tolerate the industrial calendaring process for electrode manufacturing, with extraordinary mechanical strength that could withstand a pressure of 200 MPa without breakdown, and exhibit only ≈40% particle expansion upon full lithiation.^[^ ^169^ ^]^

Although the Si/C superstructure obtained by the superparticles assembly strategy can effectively solve the defects of silicon‐based materials and improve the corresponding electrochemical performance of electrodes. However, the contact between silicon and carbon or other the adjacent conductive medium is almost always via single‐ or few‐ point physical binding, which hinders the rapid ions as well as electrons transport from and to silicon to a certain extent, affecting the rate performance and cycle stability. To solve the contact interface defect between silicon and conductive medium, a 2D covalent encapsulation strategy was proposed to establish an effective and robust electrical contact interface between silicon nanoplates and conductive medium through a 2D covalent bonding (interfacial Si‐O‐C bonds), thereby realizing the rapid ions and electrons transport from and to silicon.^[^ ^170^ ^]^ The subsequently assembled silicene flowers/graphene (SF@G) materials with microsized hydrangea flower‐like structure composed of many interconnected nanoplates (Figure 7f). Based on the chemical composition and the interface morphology of the material, this unique skin‐like bonding design greatly changed the interface between the silicon‐based material and the electrolyte, maintaining structural stability during the charging and discharging process, which endowed the electrode with stable high‐rate and high‐capacity lithium storage capacity, resulting in an excellent level of integrated performances.

### 3.5 Interconnected Assembly

**Figure 8:**
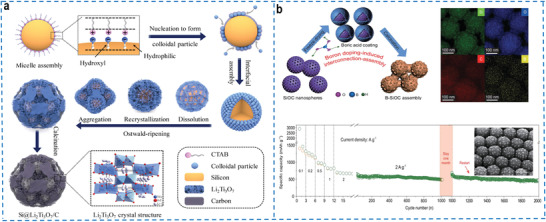
a) Schematic illustration for the preparation of H‐SiLC. Reproduced with permission.^[^ ^173^ ^]^ Copyright 2022, Elsevier. b) Schematic illustration of the boron doping‐induced interconnection‐assembly process, the corresponding elemental mapping of the B‐SiOC assembly, and rate and cycling performance of B‐SiOC anode. Reproduced with permission.^[^ ^174^ ^]^ Copyright 2020, Oxford University Press on behalf of China Science Publishing and Media.

Based on various superstructure assembly strategies, silicon‐based assemblies with different morphologies and functions from the simple nanoscale to complex hierarchical structures have been successfully synthesized, such as pomegranate‐like Si/C microsphere, hierarchical structure Si@TiO_2_@C and other superstructures, which effectively solve the defects of low conductivity, low tap density, large volume expansion, and low thermodynamic stability.^[^ ^166^, ^167^, ^168^, ^171^, ^172^ ^]^ However, there are weak interactions between the building blocks inside the superstructures prepared by the vast majority of strategies, even just a simple stacking of building blocks induced by various driving forces through steps such as coating, spray drying and CVD, etc., which may bring about structural damage and affect the overall structural stability of the electrode, resulting in a sharp capacity decay and unstable cycling performance. By introducing an interconnected assembly strategy, a connection module is constructed between the building blocks to achieve a substantial connection, thereby improving the interfacial stability of the assembly. For example, Shi et al. proposed an interfacial assembly strategy with “nucleation‐assembly‐dissolution‐growth”, and assembled nanosheet structured Li_2_Ti_3_O_7_ on the surface of Si nanoparticles (Si@Li_2_Ti_3_O_7_) to improve the lithium ions diffusion capacity. As shown in **Figure** **8**a, then more and more 2D nanosheets were generated as the Ostwald‐ripening progresses, which accelerated the interconnection between Si@Li_2_Ti_3_O_7_ particles to form a hierarchical Si@Li_2_Ti_3_O_7_/C microcluster (H‐SiLC), showing excellent cycle stability.^[^ ^173^ ^]^ Although this nanosheet connection module can promote the interfacial contact between the primitives, it still fails to provide a robust connection. To solve this problem, a novel boron doping‐induced interconnection assembly (BDIIA) method was proposed. Porous SiOC nanospheres with carbon matrix homogeneously distribution at the atomic scale were induced by boric acid to construct robust boron‐doped SiOC interconnect assemblies (B‐SiOC) (Figure 8b), which consisted of rough‐surfaced, interconnected, and strongly coupled nanospheres.^[^ ^174^ ^]^ The explored melt‐etching and nucleation‐growth mechanism demonstrated the formation of a flowing interface layer composed of boron oxide, SiOx and carbon at elevated temperature, which induced dense packing of SiOC nanoparticles. In particular, the interfacial composition of the initial SiOC particles was crucial for the boron‐induced interconnection assembly and surface roughening. During the nucleation and recrystallization, the interconnected “neck”‐like links were created between adjacent nanospheres. More interestingly, this “neck”‐like link structure still maintained the original robust interfacial connection after long‐term cycling, which significantly reduced the interparticle resistance and achieved high interfacial stability of the assembly structure. Moreover, this robust and stable interfacial connection was beneficial to provide a continuous electronic network, endowing the electrode with excellent high‐rate capacity and unparalleled long‐term cycle life stability.

## 4 Summary and Perspective

**Figure 9:**
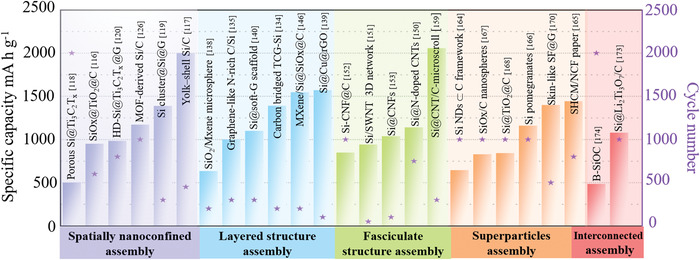
The electrochemical performance of the reported various silicon‐based assembled anodes.^[^ ^116^, ^117^, ^118^, ^119^, ^120^, ^126^, ^134^, ^135^, ^138^, ^139^, ^140^, ^146^, ^150^, ^151^, ^152^, ^153^, ^159^, ^164^, ^165^, ^166^, ^167^, ^168^, ^170^, ^173^, ^174^ ^]^

**Figure 10:**
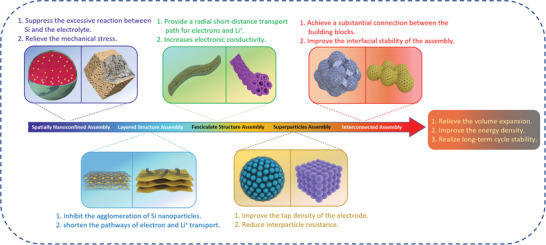
Summary of the assembly methods and main characteristics of silicon‐based assembly anode.

To construct silicon‐based anodes for lithium‐ion batteries with high energy density and excellent electrochemical performance, novel energy storage devices with different morphologies and functions can be prepared by assembly strategies based on traditional materials science and chemical reaction engineering. The behavior of assembly is mainly realized based on the interaction force of non‐covalent bonds, as an effective strategy to optimize the performance of silicon‐based anode, which is of great significance for the electrochemical performance of lithium‐ion batteries systems. When the silicon‐based assembly is used as the anode electrode of the lithium‐ion battery, the energy storage and conversion function of the electrode can be better improved: 1) high bulk density endows the electrode with excellent volumetric specific capacity; 2) the close‐packed assembly framework can not only effectively shorten the transfer pathways of electrons and ions and improve the reaction kinetics of the system, but also reduce the interparticle resistance of the nanosphere and improve the overall energy density of the lithium‐ion battery; 3) low electrode interfacial area can avoid side reactions with the electrolyte, thereby obtaining higher Coulombic efficiency; 4) the strong hierarchical structure that can always maintain structural integrity during cycling, which is conducive to stable, fast, and high‐capacity lithium storage performance. More importantly, the assembly of silicon‐based anodes is highly versatile and diverse. This review highlighted the construction methods, various driving forces, influencing factors and functions of the assembly. In addition, the latest research progress of silicon‐based assembled anodes is systematically summarized according to different assembly strategies. The electrochemical performance, assembly methods, and main characteristics of silicon‐based assembly anodes for the reported various silicon‐based assembled anodes are summarized in **Figures** **9** and **10**, respectively, revealing that the construction of multistage complex structures is an effective method to solve the defects of silicon anodes. All assembly strategies have unique advantages, which are very promising in the development of high‐performance silicon‐based anode materials. The synergistic use of multiple strategies can better present the advantages of the assembled structure. It is difficult to determine which approach is most advantageous. The functions and advantages of the assembly strategies will be continuously improved and explored through the unremitting efforts of researchers in the future.

The silicon‐based assembled structure has great potential in making significant contributions to constructing silicon anodes for lithium‐ion batteries with high energy density and excellent electrochemical performance. However, the assembly strategy is not a panacea, and its application in the battery field is still in its infancy. Considering all practical aspects, there are still many challenges to be overcome in silicon‐based assembled anodes. Therefore, the following prospects are proposed for the future development of silicon‐based assembled anodes for the lithium‐ion battery: 1) Although the assembly of silicon‐based anode materials can effectively improve the electrochemical performance, the corresponding process is relatively complicated and the cost is high, which inevitably limits its large‐scale practical production. Therefore, a simple and cost‐effective assembly process should be advocated. 2) The structural evolution of complex structures during repeated charging and discharging is critical to their actual performance, and the working conditions of most electrodes are far from the operating environments of actual equipment, so new characterization techniques need to be introduced to overcome such limitations. The introduction of in situ characterization during the exploration can not only provide the structural variation of silicon‐based assemblies but also offer valuable guidance for the construction process. 3) The assembly process is difficult to be completely and precisely controlled, which is a technical bottleneck of designing and fabricating the assembly with ideal function and morphology, so the assembly technology with high controllability is urgently needed. It is advocated to clarify the interface properties and reaction mechanisms of various silicon‐based structures, to achieve targeted assembly and integration of different assembly structures. Assembly, as a tool capable of precisely controlling the interfacial chemical structure, is considered to play a crucial role in constructing novel energy storage devices and improving electrochemical performance. It is hoped that the great potential of assembly structure can be fully realized in future research on the science and technology of advanced materials.

## Conflict of Interest

The authors declare no conflict of interest.
